# Physical Performance Impairment with Cannabis Consumption in Adults Over 12 Hours

**DOI:** 10.1177/00472379261433203

**Published:** 2026-03-23

**Authors:** Waseem Abu-Ashour, Michael Wahl, Saman Hadjizadeh Anvar, Mohammadmahdi Bahrami, Ali Zahiri, Jose Carlos Aragão-Santos, John T. Weber, David G. Behm

**Affiliations:** 1 School of Pharmacy, 7512 Memorial University of Newfoundland, St. John's, Newfoundland and Labrador, Canada; 2 Faculty of Medicine, 7512 Memorial University of Newfoundland, St. John's, Newfoundland and Labrador, Canada; 3 School of Human Kinetics and Recreation, 7512 Memorial University of Newfoundland, St. John's, Newfoundland and Labrador, Canada; 4 Graduate Program in Health Sciences, 735598 Federal University of Sergipe, Aracaju/SE, Brazil

**Keywords:** Marijuana, intoxication, endurance, motor control, health policy, cannabis testing

## Abstract

Assessing cannabis-induced impairment in physical performance is critical for safety-sensitive occupations. This study evaluated the magnitude and duration of impairment following acute cannabis use. Twenty-eight adult users completed baseline and follow-up assessments at 1, 6, and 12 h after smoking a standardized cannabis cigarette. Measures included subjective intoxication, vital signs, psychomotor performance, muscle strength, balance, and endurance. Blood Δ9-tetrahydrocannabinol (THC) concentrations peaked at one hour and declined by six hours (*p* < 0.001), while Carboxy-THC showed minimal temporal variation (*p* = 0.005). Cannabis use significantly increased systolic blood pressure and heart rate, elevated muscle force variability, reduced the rate of force development, and impaired balance and endurance for up to 12 h. Despite reduced perceived intoxication, measurable functional impairments persisted, indicating prolonged performance effects.

Public Health Significance Statement:

This study shows that smoking cannabis can affect balance, muscle control, and endurance for up to 12 h, even when people no longer feel “high.” These lingering effects could put workers and the public at risk in jobs that require alertness, quick reactions, or physical precision, such as construction, transportation, or emergency response. The findings highlight the need for workplace policies that recognize how long cannabis can impair performance and help protect both employees and community safety.

## Introduction

Cannabis consumption has risen substantially globally following legalization movements, including Canada's 2018 Cannabis Act (Rotermann, 2019). Usage has increased across demographics, with particularly high prevalence among working-age adults and male individuals, especially those aged 18–24 (Rotermann, 2019). Importantly, cannabis use is not limited to off-duty periods: national survey data indicate that approximately 26% of Canadians who use cannabis report consumption during work hours or immediately prior to work (Health Canada, 2018), highlighting the potential for exposure during safety-relevant occupational activities (Frone and Trinidad, 2012). This trend spans North America and Europe (Rotermann, 2019; European Monitoring Centre for Drugs and Drug Addiction, 2019; Hasin et al., 2019), raising concerns about cannabis exposure during periods when individuals may be engaged in occupational tasks that require physical coordination, vigilance, and rapid decision-making, thereby making understanding workplace impairment crucial (Frone and Trinidad, 2012; National Academies of Sciences, Engineering and Medicine, 2017).

Evidence on workplace injuries and cannabis shows mixed results, with some studies indicating higher risk (Els and Straube, 2016; Carnide et al., 2023; Li et al., 2024; WHO, 2016) and others finding no connection (Biasutti et al., 2020; Zhang et al., 2020). For example, longitudinal survey data suggest that cannabis use before or during work is associated with an increased risk of subsequent self-reported workplace injury (Carnide et al., 2023), while quasi-experimental policy analyses have linked cannabis legalization and retail expansion to higher injury rates among younger workers in population-level labor statistics (Li et al., 2024). In contrast, studies reporting null findings often rely on cross-sectional designs, broad past-year exposure measures, and self-reported injuries without information on timing of use relative to work or objective impairment assessment (Zhang et al., 2020). These inconsistent findings complicate policy development and risk management strategies, particularly in safety-sensitive industries. Systematic reviews highlight that much of the occupational literature lacks direct assessment of impairment, limiting the ability to distinguish cannabis exposure from functional risk (Biasutti et al., 2020). Despite these limitations, random testing programs in safety-sensitive positions continue to identify a substantial proportion of employees testing positive for cannabis, underscoring the relevance of impairment-focused research (TTC, 2019). From a workplace safety perspective, this disconnect between biological detection and functional impairment has been noted as a critical challenge for evidence-based policy development (Els and Straube, 2016).

Cannabis may impair attention, concentration, and memory among frequent and non-frequent users (Crane et al., 2013). Evidence syntheses indicate that episodic memory impairment is among the most consistently reported neurocognitive effects, with additional deficits observed across executive function, attention, and psychomotor domains depending on dose, task demands, and user characteristics (Crane et al., 2013). Impairments occur following both small and large doses of delta-9-tetrahydrocannabinol (THC) (Crane et al., 2013). THC, the primary psychoactive component, binds to cannabinoid receptors, affecting psychomotor and cognitive functions (McLaughlin et al., 2000; Zou and Kumar, 2018). Controlled experimental studies further demonstrate that short term effects of THC inhalation increase premature responding and slow task execution time, even when accuracy on some higher-order cognitive tasks appears relatively preserved, indicating that impairment may manifest as slowed performance or reduced response control rather than overt errors alone (Hart et al., 2001). These neurophysiological effects provide a plausible biological basis for impaired task performance, particularly in roles that depend on rapid motor responses, sustained force production, balance, and coordination (McLaughlin et al., 2000; Karila et al., 2014; Hart et al., 2001).

Mechanistic evidence also suggests that THC can disrupt motor execution at the level of the force–time trajectory, including reductions in peak force and rate of force development (RFD), which is directly relevant to occupational tasks requiring rapid force generation and precise force control (McLaughlin et al., 2000). In contrast, cannabinoids like CBD (cannabidiol) and CBN (cannabinol) do not cause direct impairment (Khalsa et al., 2022). Cannabis-related impairments such as reduced concentration, slower reactions, impaired memory, and diminished coordination which decrease efficiency and increase errors in safety-sensitive occupations (Bernerth and Walker, 2020; Hazle et al., 2022). This is particularly relevant for construction, transportation, and emergency response occupations, which demand precision, balance, rapid force generation, and sound decision-making (Beus et al., 2016; Cornelissen et al., 2017). These dose-dependent impairments increase accident risks, especially with fatigue or high workload (Cornelissen et al., 2017).

Consistent with this concern, a substantial body of research demonstrates that cannabis adversely affects driving performance through impaired motor skills (Bondallaz et al., 2016; Ramaekers et al., 2006; McCartney et al., 2021; Preuss et al., 2021), serving as an established analogue for other complex, safety-critical occupational tasks (Bondallaz et al., 2016). Driving is widely used as a model of safety-critical performance because it requires the real-time integration of perception, psychomotor control, executive functioning, divided attention, and inhibitory control under dynamic conditions that closely resemble many occupational tasks (Bondallaz et al., 2016; McCartney et al., 2021; Preuss et al., 2021). Experimental studies using controlled THC exposure demonstrate impairments in driving-relevant domains such as psychomotor control, executive planning, and inhibitory motor control, with effects persisting for up to six hours following high-potency cannabis consumption (Ramaekers et al., 2006). These impairments show dose–response relationships and are not fully mitigated by compensatory behaviors, particularly under increased task complexity or unexpected events (Bondallaz et al., 2016; McCartney et al., 2021). Quantitative syntheses further indicate that while many driving-related cognitive skills recover within approximately 5–7 h following moderate inhaled doses, substantial heterogeneity remains by dose, route of administration, and user frequency, with oral formulations often producing longer-lasting impairment (McCartney et al., 2021). Together, these findings provide a rationale for examining impairment beyond short “peak intoxication” windows and motivate extended observation periods when evaluating work-relevant performance.

Despite existing research, important gaps remain regarding the duration and functional relevance of cannabis-related impairment, particularly beyond the acute intoxication period. While immediate effects are well documented, residual impairments vary substantially across individuals and task domains (Bolla et al., 2002). Prior research demonstrates pronounced interindividual variability driven by dose, frequency of use, and tolerance, complicating efforts to define uniform impairment or recovery timelines relevant for return-to-work decisions (Bolla et al., 2002; Schreiner et al., 2012). Evidence from heavy-use cohorts suggests dose-related decrements in psychomotor speed and manual dexterity that may extend beyond acute intoxication; however, the current scientific literature does not consistently support persistent impairments in memory and executive functioning after prolonged abstinence, while meta-analytic findings indicate that many residual effects resolve after prolonged abstinence (Bolla et al., 2002; Schreiner et al., 2012). Importantly, tolerance in frequent users may attenuate observable impairments on low-demand tasks, potentially masking functionally meaningful deficits under higher cognitive or physical load (Heishman et al., 1990). From a policy perspective, this variability undermines one-size-fits-all guidance based solely on time since use, biological thresholds, or subjective intoxication.

Accordingly, the present study aimed to investigate the degree and duration of impairment associated with cannabis use on physical and cognitive performance in adults, with assessments conducted immediately after consumption and up to 12 h post-use. By extending evaluation beyond commonly studied acute windows and focusing on objective, performance-based outcomes directly relevant to occupational demands, this study seeks to address critical gaps in the evidence base needed to inform workplace safety policies, impairment education, and risk-mitigation strategies.

## Methods

Based on an “a priori” statistical analysis (G*power version 3.1.9.2, Dusseldorf Germany) to obtain a small magnitude effect size, approximately 18 adult participants were needed to achieve an effect size of 0.3, considering the interaction between two groups and four times, alpha (α) of 0.05 and a power of 0.8. Through convenience sampling, 28 participants (Experimental group: 15 frequent cannabis users and 13 non-smoking controls) were recruited (Table 1). Frequent cannabis consumers were defined as those smoking or ingesting edibles three or more times weekly over the last six months. For comparison, McCartney et al. (2021) define regular cannabis consumers as individuals who use cannabis weekly or more. Participants were recruited through advertisements on social media platforms, local media outlets, posters displayed on a university campus, and word-of-mouth referrals. The study did not employ randomization to cannabis exposure. Group assignment was intentional and based on participants’ self-reported cannabis use patterns rather than randomization. Frequent cannabis users were assigned to the experimental group and received the cannabis intervention, while participants assigned to the control group did not consume cannabis on the testing day.

**Table 1.** Participants’ Characteristics.

Characteristics	Control (13)	Experimental (15)	Total (28)	p-Value
Age (years)	32.3 (12.5)	32.5 (8.2)	32.4 (10.2)	0.968
Height (m)	1.72 (0.08)	1.77 (0.09)	1.75 (0.09)	0.179
Weight (Kg)	83.2 (18.0)	83.6 (18.1)	83.4 (17.7)	0.959
BMI (Kg/m^2^)	28.2 (5.6)	26.7 (5.4)	27.4 (5.4)	0.476
Categorical variables				
Sex (M/F)	9/4	12/3	21/7	0.512
Physical activity level: Absolute number (relative %)				
No regular activity	0 (0.0)	1 (3.6)	1 (3.6)	0.631
2-3 times / week	8 (28.6)	9 (32.1)	17 (60.7)	
≥ 5 times / week	5 (17.9)	5 (17.9)	10 (35.7)	
Average cannabis use (absolute/relative)				
No use	8 (28.6)	0 (0.0)	8 (28.6)	**0**.**012**
1 time / month	1 (3.6)	0 (0.0)	1 (3.6)	
1 time / week	1 (3.6)	1 (3.6)	2 (7.1)	
1 time / year	1 (3.6)	0 (0.0)	1 (3.6)	
3 times / year	1 (3.6)	0 (0.0)	1 (3.6)	
≥ 3 times / week	1 (3.6)	14 (50.0)	15 (53.6)	

*Note.* P-values for the continuous variables based on independent t-test and chi-squared test for the categorical variables.

Exclusion criteria included: history of neurological or psychiatric disorders, engagement in psychological treatment (within previous 6 months), current or past diagnoses of psychotic disorders, participants taking medications that could influence psychometric testing, history of alcohol or other substance use disorder (other than cannabis use disorder), and pregnant, looking to become pregnant, or breastfeeding female participants. Participants refrained from consuming cannabis 12 h before testing and during the entire testing day. The experimental protocol and consent form was emailed initially and verbally explained upon arrival. All data was anonymized. This research was approved by the institution's Human Research Ethics Board and conducted according to the Declaration of Helsinki. Detailed methods found in Supplementary Material.

### Experimental Design

**Figure 1.**
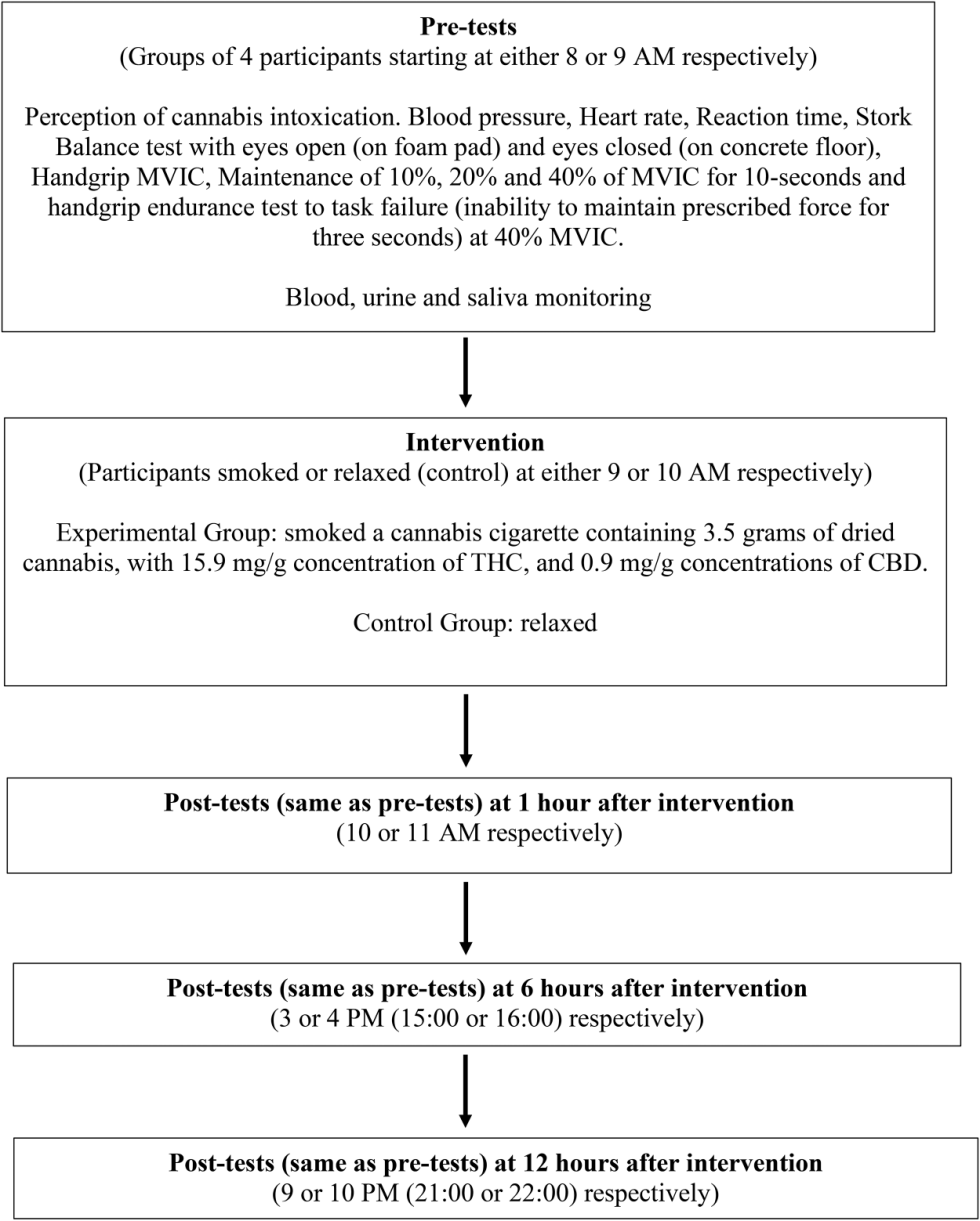
Experimental design.

Testing occurred over a three-day period with each participant involved for a 14-h period within one day (Figure 1). Testing commenced at either 8 or 9 AM with groups of four individuals per session. Testing prior to intervention (cannabis smoking at 9 or 10 AM) and post-intervention (at 1-, 6- and 12-h post-smoking) involved measures of body mass, height, perceived feelings of intoxication, resting blood pressure (BP) and heart rate (HR), reaction time (RT), stork balance test on a padded mat with eyes open and on concrete floor with eyes closed, dominant limb handgrip maximal voluntary isometric contraction (MVIC) strength, maintenance of 10%, 20% and 40% of handgrip MVIC for 10-s, and handgrip endurance test to task failure at 40% MVIC. These measures are crucial in various workplaces where slower reaction times, poor balance, impaired strength or endurance could negatively impact work performance or contribute to injuries. Blood, urine, and saliva collection was conducted by certified nurses at each testing period. The control group served as a non-intoxicated comparison condition and did not receive cannabis, regardless of past or infrequent use history.

### Independent Variable (Cannabis Intervention)

Following pre-tests, participants smoked a cannabis cigarette (Ezee Chill pre-roll: God Father OG) provided by Atlantic Cultivation Inc. According to the manufacturer website (https://www.leafly.ca/strains/godfather-og), this indica strain generally produces sedating and relaxing effects. The pre-rolled cigarette contained 0.35 grams of dried cannabis, with 15.9 mg/g concentration of THC, and 0.9 mg/g concentrations of CBD. Participants consumed the entire product within 10–20 min to ensure consistency. Cannabis administration was restricted to participants in the experimental group who were frequent users. All participants reported familiarity with smoked cannabis, and as frequent consumers, the amount was within their normal consumption range. Smoking was monitored by a researcher to ensure full inhalation and observe for adverse reactions (none occurred). Two trained registered nurses were on site for monitoring. After consumption, participants returned to the testing site for remaining testing periods.

### Dependent Variables (Measures)

#### Anthropometric Data

Body mass was measured using a calibrated commercial scale (Accuweight Moreno Valley, CA 92551, USA), calibrated to zero before each session using standard weights, with ±0.1 kg precision. Height was measured with a commercially available measuring tape secured to a vertical wall, verified against a standardized metal rod prior to the study and re-verified weekly, recorded to the nearest 0.1 cm. Participants stood upright without footwear, heels together, and head positioned back to the wall. Participants reported age, sex, and activity level, defined as either sedentary, recreationally active, or trained (McKay et al., 2022). These measures were critical for understanding potential baseline differences, as body mass can influence cannabinoid metabolism (Rossi et al., 2018; Lucas et al., 2018) and activity level could moderate performance outcomes.

#### BP & HR

BP and HR were monitored as indicators of increased stress that could adversely affect health (Liu et al., 2017; Virginia de Carvalho, 2013). Participants remained seated and relaxed for 2 min before measurement with an Omron device (Omron Healthcare, Inc. Tokyo). The cuff was placed on the left arm superior to the cubital fossa with the arm relaxed, extended, palm supinated, and resting on a table at shoulder height (Brady et al., 2021).

#### Subjective Perceived Feelings of Intoxication

Participants marked a 10 cm horizontal line (0–100%) indicating how “high” or “stoned” they felt, with zero being no intoxication and 100% being the most intoxicated they had ever felt.

#### Reaction Time (RT)

RT was measured on a laptop using a web application (faculty.washington.edu/chudler/java/redgreen.html). Participants tapped the trackpad when a green light appeared. Five attempts were averaged, with outliers excluded. Day-to-day reliability using intraclass correlation coefficient (ICC) has been reported between 0.79 and 0.93 (Behm et al., 2004).

#### Static Stork Balance Test

Two trials of the Static Stork balance test were conducted with eyes open and eyes closed. For eyes open, participants stood on a 45 cm^2^ foam pad (10 cm thick). For eyes closed, they stood on concrete floor. Participants placed hands on hips and positioned the non-supporting foot against the inside knee of the supporting leg after one minute of practice. Timing stopped if hands came off hips, supporting foot moved, or non-supporting foot lost contact with the knee. The longest time from two trials on both legs was analyzed. Reliability has been reported with ICCs ranging from 0.91 to 0.93 (Aranha et al., 2016).

#### Handgrip Maximum Voluntary Isometric Contractions (MVIC)

While standing, participants gripped a custom-designed isometric handgrip device with their dominant hand, performing two handgrip MVICs with a third if the second exceeded the first by >5%. One-minute recovery was provided between attempts. The steel bar grip was attached to a Wheatstone bridge strain gauge (Omega Engineering Inc., LCCA 250). During 4-s MVICs, participants squeezed as hard and fast as possible with the arm abducted to prevent bracing. Peak force and rate of force development from 0–50, 50–100, 100–150, and 150–200 ms were recorded. Forces were amplified (500x) (Biopac Systems Inc., DA 150), converted (MP150WSW), and monitored at 2000 Hz sampling rate. This laboratory has reported excellent intersession reliability (ICCs of 0.98–0.99) using these techniques (Behm et al., 1996, ., 2002; Low et al., 2019).

#### Movement (Submaximal Force) Control

Two minutes after MVIC, participants maintained relative forces of 10%, 20%, and 40% of MVIC for 10-s each while viewing a computer monitor. One-minute recovery was provided between contractions. Data from the middle 6-s was analyzed for average relative deviation from prescribed force and standard deviation as a measure of force output variability. Prior research has reported day-to-day reliability (ICC) of 0.8, with between-test reliability of 0.88 (Behm et al., 2004).

#### Handgrip Endurance

The 40% handgrip MVIC was maintained until task failure, defined as inability to maintain the prescribed force for 3-s (second occurrence). Maximum endurance time was recorded.

#### Electromyography (EMG)

Surface EMG recorded forearm and finger flexor activity. Before electrode placement, skin was shaved, abraded, and cleaned with isopropyl alcohol. Self-adhesive Cl/AgCl bipolar electrodes (MeditraceTM 130) were placed according to SENIAM guidelines (Hermens et al., 1999) at 80% of the distance from radial head to medial epicondyle and 80% of lateral to medial side distance of the palmar forearm. EMG signals were amplified 1000x, filtered (10–500 Hz), and sampled at 2000 Hz. EMG integral and root mean square were measured over a 1-s window around peak force. Median frequency was obtained from 5–250 Hz. These parameters were monitored during movement control contractions and analyzed for each quartile of the endurance protocol.

### Cannabinoid Testing

THC and Carboxy-THC levels were measured in blood and urine samples at baseline and at 1-, 6-, and 12-h post-consumption to understand pharmacokinetic profiles (Huestis and Smith, 2018). A registered nurse collected samples, which were stored at −80°C until shipping.

#### Blood Cannabinoids Lab Processing

Extraction and analysis was performed by Dynacare labs (Ottawa, Ontario, Canada). Blood samples were collected using BD Vacutainer^®^ K2 EDTA 4 ml tubes. Samples (100 µL) were mixed with methanol containing Cannabinoids Working Internal standard, vortexed for 60 s, equilibrated at room temperature for 10 min, then centrifuged at 4500 RPM for 5 min. Supernatant was analyzed using Prominence HPLC System (Shimadzu) and 6500+ QTRAP LC-MS/MS (SCIEX). The limit of quantitation was 2 ng/mL, with an analytical range of 2.0 to 5000 ng/mL.

#### Urine Cannabinoids Lab Processing

Urine samples (100 µL) with Cannabinoids Working internal Standard were hydrolyzed using enzyme and alkaline digestion, neutralized, and vortexed for 60 s before analysis as described above.

#### Saliva Test

The VeriCheck Oral Fluid THC Test was used to measure THC levels in saliva, designed to detect concentrations as low as 10 ng/mL (https://www.drugtestkits.ca/vericheck-oral-fluid-saliva-thc-test).

### Statistical Analyses

Data were analyzed using Jamovi (Version 2.3.18.0). Continuous characterization data were presented with marginal means and standard deviations and compared using one-way ANOVA. Categorical data were shown based on absolute and relative frequency and compared using chi-square test. For physical performance measures, generalized linear mixed models with Gamma distribution were used. Group, time, and interaction effect were defined as fixed effects, with participants’ intercepts as random effects. When interaction effects were detected, estimates were analyzed using pre-test and control group as reference levels, with significance at *p* < 0.05.

THC metabolite concentrations were analyzed using two-way ANOVA with ‘time post-consumption’ as within-subject factor and ‘treatment’ as between-subject factor. Sphericity was verified using Mauchly's test, with Greenhouse-Geisser corrections applied when necessary. Post hoc comparisons used Bonferroni correction, with significance at *p* < 0.05.

## Results

Among the 28 participants (15 experimental, 13 control), only average cannabis consumption differed significantly between groups (*p* = 0.012), with higher use in the experimental group (Table 1).

### Intoxication, HR, and BP

**Figure 2.**
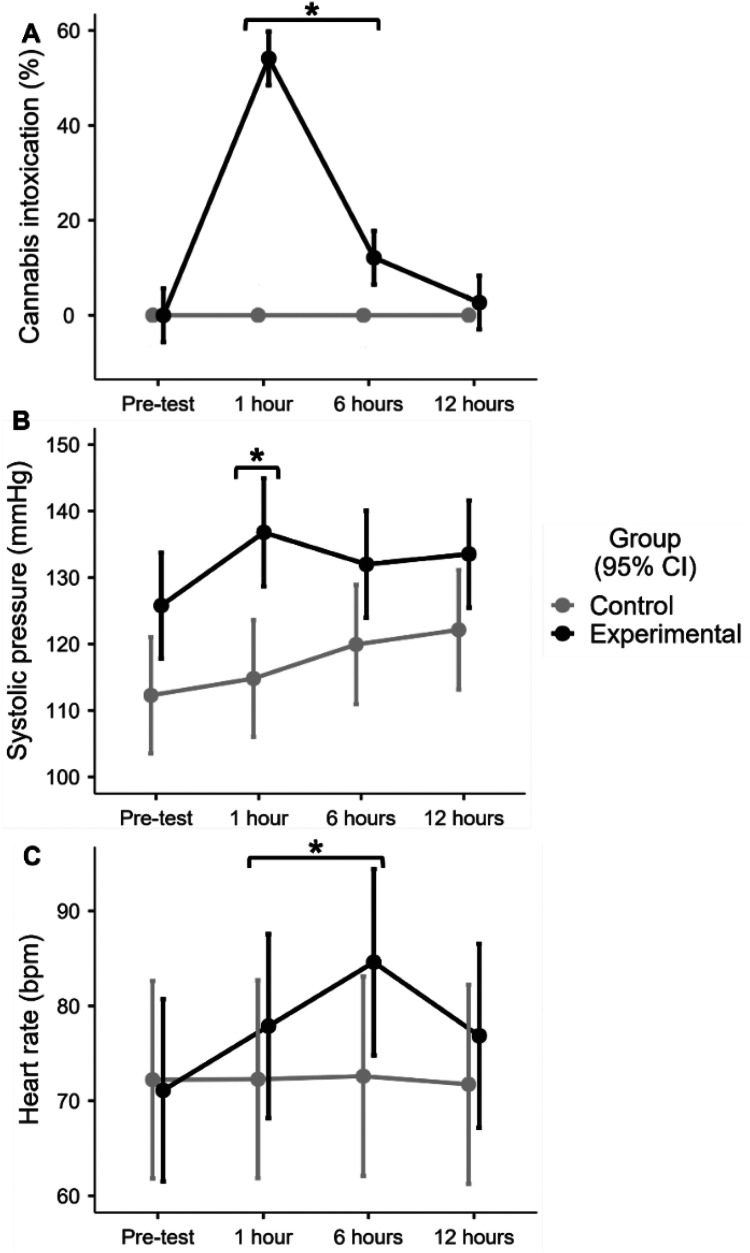
Means and 95% confidence intervals for the perception of cannabis intoxication (A), systolic pressure (B), and heart rate (C) at all times were evaluated for the control (grey line) and experimental (black line) groups. *Note.* “*” Indicates a p-value smaller than 0.05 for the interaction effect based on the variation from the pre-test compared between groups.

There were interaction effects for the cannabis intoxication perception (χ^2^_(3)_ = 19.20; *p* < 0.001), systolic BP (χ^2^_(3)_ = 9.30; *p* = 0.026), and HR (χ^2^_(3)_ = 15.62; *p* = 0.001). On average, the experimental group perceived their extent of intoxication to be rated at 53.0, 13.0, and 2.8, at 1- (95% CI = 43.84–63.84; *p* < 0.001), 6- (95% CI = 6.43–16.67; *p* = 0.022), and 12-h respectively (95% CI = -1.08–5.93; *p* = 0.175) (Figure 2A). The systolic BP was 8.5 mmHg (95% CI = 1.04–15.93; *p* = 0.026) higher in the experimental vs. the control group one hour after smoking but not at 6- or 12-h (Figure 2B). The HR was higher both at 1- (β = 6.71; 95% CI = 0.43–12.99; *p* = 0.036) and 6-h (β = 13.12; 95% CI = 6.59–19.65; *p* < 0.001) (Figure 2C).

### Muscle Strength and Endurance

**Figure 3.**
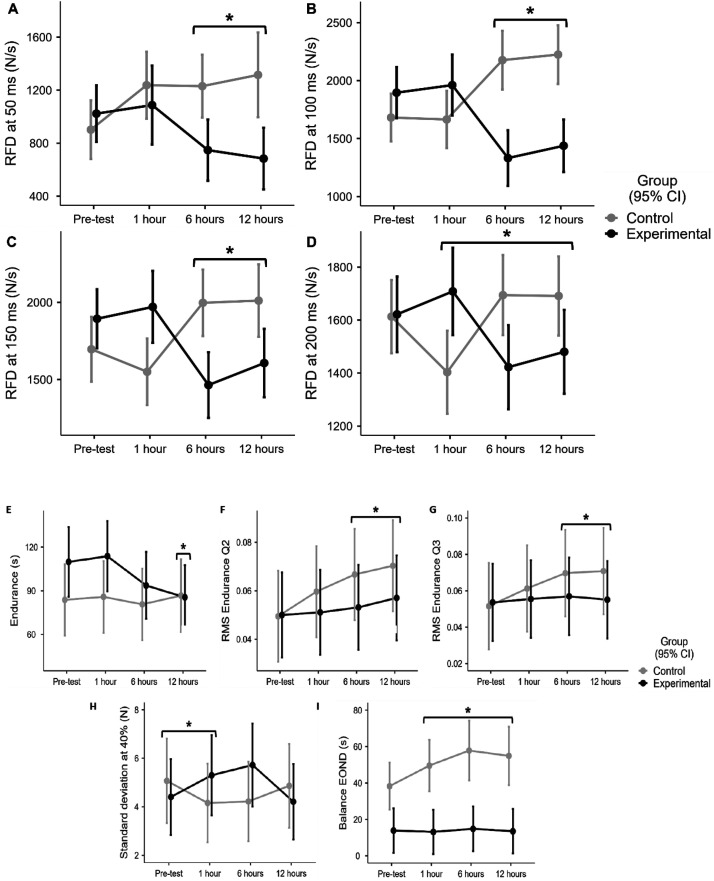
Means and 95% confidence intervals for the rate of force development (RFD) at 50 (A), 100 (B), 150 (C), 200 (D) ms at all measured times for the control (grey line) and experimental (black line) groups. *Note.* “*” Indicates a p-value smaller than 0.05 for the interaction effect based on the variation from the pre-test compared between groups. Means and 95% confidence intervals for the endurance (E), EMG root mean square (RMS) at second (F), third (G) quartile of the endurance test at 40% of maximal voluntary isometric contraction (MVIC), force variability (as measured by standard deviation of force) at 40% at 40% of MVIC (H), and balance with eyes opened with non-dominant leg (EOND) (I) at all measured times for the control (grey line) and experimental (black line) groups. *Note.* “*” Indicates a p-value smaller than 0.05 for the interaction effect based on the variation from the pre-test compared between groups.

The single 4-s MVIC force or EMG activity had no main effects or interactions. Regarding RFD, there was an interaction effect for all the testing times (χ^2^_(3)_ = 16.00–221.14; *p* < 0.001). For the RFD at 50, 100, and 150 ms, the experimental group showed a reduction at 6- (β_50ms_ = −604.1; 95% CI = −789—−419 / β_100ms_ = −1059.3; 95% CI = −1241—−877/ β_150ms_ = −730.0; 95% CI = −890—−569 / *p* < 0.001) and 12-h (β_50ms_ = 752.6; 95% CI = −1097.6—−407.5 / β_100ms_ = −1001.3; 95% CI = −1169—−833 / β_150ms_ = −601.7; 95% CI = −773—−430 / *p* < 0.001) after smoking compared to the control group (Figures 3A, 3B, and 3C, respectively). While at 200 ms, the experimental group showed an increase after 1-h (β = 296.5; *p* < 0.001) and reductions after 6- (β = −280.3; *p* < 0.001) and 12-h (β = −219.4; *p* < 0.001) (Figure 3D).

There was an interaction effect for the endurance test duration (χ^2^_(3)_ = 10.42; *p* = 0.015), with the experimental group sustaining the 40% MVIC for 27.3-s (95% CI = −47.51—−7.08) less than the control group at 12 h after cannabis smoking (Figure 3E). Muscle activity, as represented by the EMG RMS, also presented a significant interaction at the second (F_(3,78)_ = 3.93; *p* = 0.011) and third quartile times of the endurance test (F_(3,75)_ = 3.90; *p* = 0.012) at 6- (β_Q2_ = −0.014; 95% CI = −0.023—−0.004 / *p* = 0.004 / β_Q3_ = −0.014; 95% CI = −0.025—−0.003 / *p* = 0.011) and 12-h (β_Q2_ = −0.013; 95% CI = −0.022—−0.004 / *p* = 0.004 / β_Q3_ = −0.017; 95% CI = −0.029—−0.006 / *p* = 0.002) after smoking with an average reduction in the EMG RMS of the experimental vs. the control groups respectively (Figures 3F and 3G).

### Muscle Force Control

A significant interaction for force variability (as measured by force SD) with 40% MVIC (χ^2^_(3)_ = 12.01; *p* = 0.007) indicated that the experimental group averaged 1.8 (95% CI = 0.29–3.32; *p* = 0.019) and 2.2 N (95% CI = 0.52–3.81; *p* = 0.010) higher SD at 1- and 6-h after smoking than the control group (Figure 3H).

### Reaction Time and Balance

RT had no significant main effects or interactions. For the balance with eyes open and the non-dominant limb, a significant interaction (χ^2^_(3)_ = 13.70; *p* = 0.003) revealed balance test duration reductions of −12.0 (95% CI = −20.88—−3.15) −18.6 (95% CI = −32.75—−4.42), and −16.9 (95% CI = −31.10—−2.82) seconds after 1-, 6-, and 12-h respectively, in the experimental group (Figure 3I).

### Cannabinoid Testing:

#### Blood THC Levels

**Figure 4.**
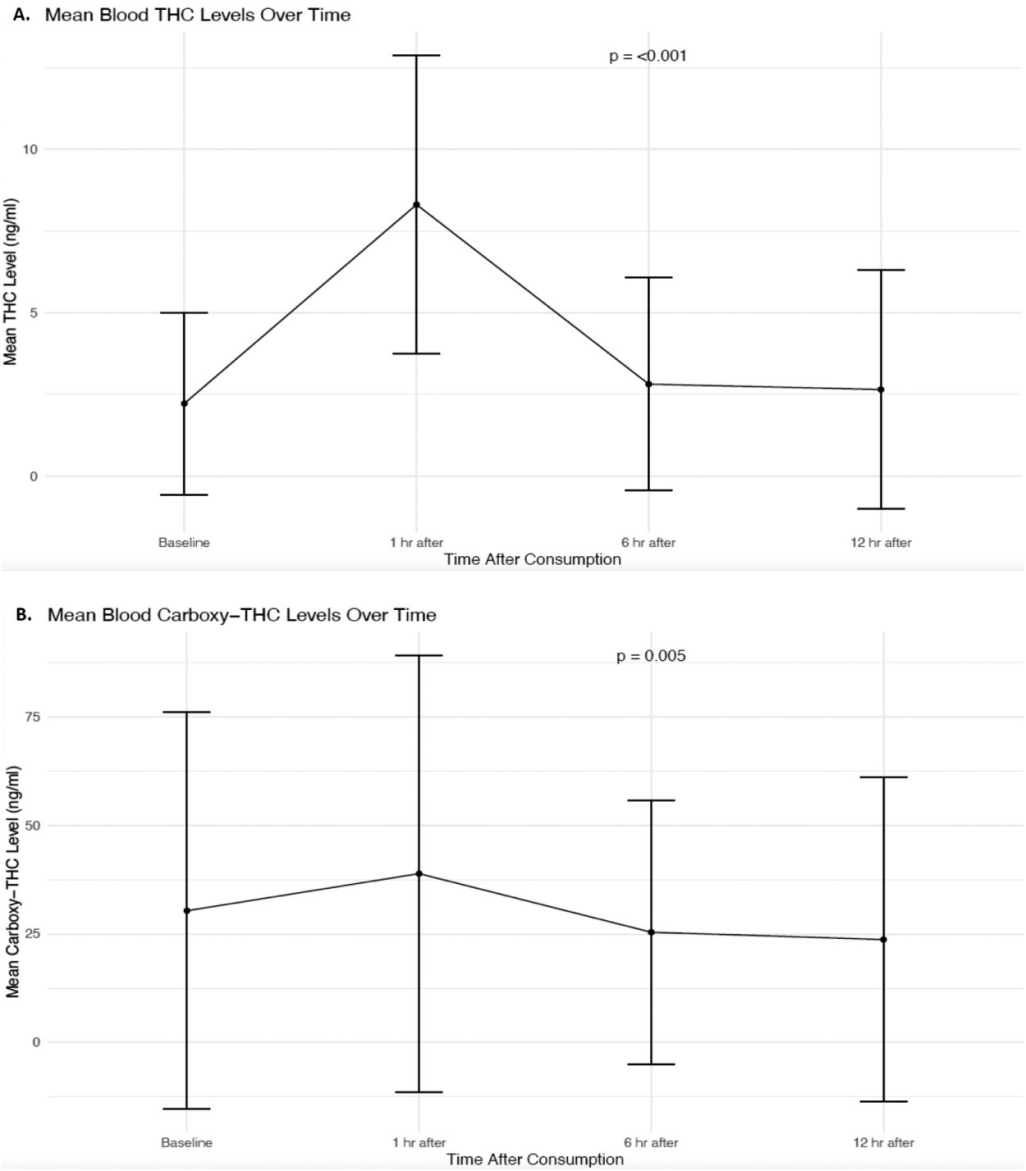
Mean blood THC levels (figure 4a) and mean blood levels of carboxy-THC (figure 4b) over time at four different time points: baseline, 1-, 6-, and 12-h after cannabis consumption. The p-values indicate that blood THC and Carboxy-THC levels respectively were significantly higher at 1-h post-consumption.

The analysis of temporal changes in blood THC concentrations across four time points relative to cannabis consumption revealed a statistically significant variation (F_(3, 39)_ = 43.983, *p* < 0.001)(Figure 4A). Post hoc analysis revealed that blood THC levels were significantly higher at 1-h post-consumption compared to baseline (*p* = 0.002). However, there were no significant differences between baseline measures versus 6- and 12-h. Moreover, blood THC at 1 h was significantly greater than at 6-h (*p* = 0.0076) and 12-h (*p* = 0.0079) with no statistically significant difference between the 6- and 12-h measures.

#### Blood Carboxy-THC Levels

The analysis of the blood Carboxy-THC concentrations across four time points relative to cannabis consumption indicated a significant variation over time (F_(3, 39)_ = 5.054, *p* = 0.005) (Figure 4B). Post hoc analysis indicated that there were no statistically significant differences in the levels of Carboxy-THC in blood between any of the time points tested.

#### Urine Carboxy-THC Levels

The investigation into the temporal dynamics of Carboxy-THC concentrations in urine revealed a non-significant trend (F_(3, 39)_ = 2.566, *p* = 0.068).

## Discussion

The study found cannabis-related physical impairments with some deficits up to 12 h after consuming one marijuana cigarette (0.35 grams of dried cannabis with 15.9 mg/g concentration of THC). Blood THC levels were significantly higher at 1-h post-consumption compared to baseline but there were no significant differences between baseline measures versus 6- and 12-h. Blood Carboxy-THC Levels followed a similar trend but did not achieve statistical significance. The hypothesis was partially confirmed as smoking cannabis increased systolic BP (1-h), HR (1- and 6-h), force variability when attempting to maintain 40% MVIC (1- and 6-h) as well as decreases in RFD (50-, 100-, 150- and 200-ms) at all force-time periods (6- and 12-h), 40% MVIC endurance time (12-h) and associated endurance task EMG (12-h) and eyes open non-dominant leg balance (1-, 6- and 12-h). Although the participants expressed minimal to no perception of intoxication at 12 h post-consumption, there were still negative physical performance manifestations.

The increase in HR (tachycardia) and BP (hypertension) are similar to prior studies. A study by Hindocha et al. (2017), participants who smoked a mixture of 66.67 mg cannabis and 311 mg tobacco were tested within an hour experienced increases in HR and diastolic BP, but no significant change in systolic BP. Another study, three experienced marijuana smokers (averaged 4.7 marijuana cigarettes per month) smoked 4 marijuana cigarettes (2 each at 9:00 and 13:00) with concentrations of 2.57% THC and were tested over 6 h on day one as well as the following day. Similar to our study, HR was elevated immediately after smoking and returned to baseline after 6 h (Heishman et al., 1990). In another study, after vaporizing and smoking cannabis containing THC doses of 0, 10, and 25 mg, HR peaked within 30-min and returned to baseline within 3- to 4-h (Spindle et al., 2018). A review by Franz and Frishman (2016), reported that marijuana cigarettes increase HR, supine systolic and diastolic BP, and forearm blood flow due to increased sympathetic nervous system activity.

There is evidence of performance impairments, with the present study highlighting difficulties with matching and maintaining a 40% MVIC force. Chung et al. (2020) demonstrated that young adults’ acute subjective marijuana “high” impaired performance with visuospatial working memory, information processing and psychomotor speed, which were related to slight decreases in cognitive functioning. Marijuana decreased the accurate detection of circular lights on the first day of testing but not the second day (Heishman et al., 1990). Cognitive and psychomotor deficits peaked at 30–60-min post-cannabis administration, not returning to baseline for 6 to 8 h in some cases (Spindle et al., 2018). Very high dosages can cause persistent, negative effects on verbal and visual memory, executive functioning, visuoperception, psychomotor speed, and manual dexterity (Bolla et al., 2002).

In terms of motor control, driving performance under the influence of cannabis has been studied. A review by Bondallaz et al. (2016) reported dose-dependent impairments in road tracking due to increased lane position variability and deficits in the ability to maintain a constant velocity. Another narrative review by Chow et al. (2019) indicated that whereas acute and infrequent cannabis use can induce cognitive and psychomotor impairment when driving, this is not consistently the case for chronic heavy use. A meta-analysis by McCartney et al. (2021) found that regular cannabis users experienced fewer driving impairments than occasional cannabis users. Their analysis suggested that individuals should wait at least 5-h following inhaled cannabis use before performing safety-sensitive tasks. Another review by Arkell et al. (2021) reported that THC impairs driving performance and can increase crash risks for up to 8-h especially with occasional users. Ogourtsova et al. (2018) found that with young recreational cannabis users, smoking a 100-mg dose of cannabis had no significant effect on simple driving-related tasks, but there were significant deficits with complex tasks, which persisted up to 5 h after use.

Within this context, the persistence of physical performance impairments observed in a predominantly frequent-use sample is notable. Despite potential tolerance effects, deficits in force control, endurance, and balance were still evident up to 12 h post-consumption, suggesting that certain neuromuscular and coordination-related outcomes may be less susceptible to tolerance than subjective intoxication or simple reaction time.

Although there was no significant effect on reaction time in the present study, RFD was adversely affected for up to 12 h post-consumption. In contrast, Hindocha et al. (2017) found a prolonged reaction time in a spatial working memory test after smoking 16.1% THC in regular consumers. Reviews also report cannabis-induced reaction time impairments in Canadian (Brubacher et al., 2020) and Muslim (Nassif, 2021) youth. However, Kvalseth (1977) had six experienced marijuana users induce 0, 6.5, and 19.5–26.0 mg THC with no effects on simple and complex reaction time but participants did experience increased error rates for the linear and rotary movements as the dose level increased. Prashad and Filbey (2017) noted that while simple reaction time often shows no deficits, tasks requiring focus and critical decision making reveal impairments.

Although cannabis-induced deficits appear to be more pronounced with more cognitively complex tasks like driving (Bolla et al., 2002; Bondallaz et al., 2016; Chung et al., 2020; Ogourtsova et al., 2018), our results reveal that activities involving either precision or maximal RFD can also be negatively impacted. Compared to low load tasks, maximal RFD involves high motor unit recruitment especially of type II motor units firing at higher frequencies and synchronization (Behm, 1995, 2004; Behm and Sale, 1993).

MVIC forces and EMG activity were also not adversely affected by the cannabis, which is partially in accord with the Burr et al. (2021) review. Only one of the four balance tests displayed a deficit. Balance is controlled by sensory and motor responses to the perceived perturbation to the equilibrium (Anderson and Behm, 2005; Ferber et al., 2002; Hrysomallis, 2011).

Exercise endurance was only adversely affected at the 12-h post-consumption period. There is scant literature on the effects of cannabis on muscular endurance. A single cannabis dose had no significant effect on exercise endurance in adults with advanced COPD (Abdallah et al., 2018). As cannabis can decrease pain (Legare et al., 2022; Pantoja-Ruiz et al., 2022), it may have offset the debilitating effects of pain on corticospinal excitability (Del et al., 2007) and perception of effort (Steele, 2020) for the first 6 h of testing. The saliva test kit we used failed to detect THC, contrasting with other studies demonstrating the utility of saliva tests (Di Ciano et al., 2023; Churcher et al., 2023; Lee et al., 2016). Regarding workplace testing, urinalysis is not recommended for identifying job safety risks, while blood testing for active THC can be considered (Macdonald et al., 2010). However, blood levels alone cannot reliably determine impairment, necessitating neurocognitive testing (Goldsmith et al., 2015).

It is important to note that participants in the experimental group reported significantly higher baseline cannabis use compared to the control group, reflecting intentional group assignment rather than randomization. This design choice was implemented to ensure participant familiarity with smoked cannabis and to minimize the risk of adverse reactions; however, it represents an important limitation when interpreting the results. Higher habitual cannabis use may confer partial tolerance to some cognitive or psychomotor effects of THC, potentially attenuating observable impairments relative to infrequent or naïve users. As a result, the magnitude of impairment observed in this study may underestimate effects that could occur in individuals with lower baseline exposure.

Additional limitations should be considered when interpreting these findings. First, the relatively small sample size, while justified given the intensive physiological and performance-based testing protocol, may limit statistical power for detecting smaller effects and reduce generalizability to broader worker populations. Second, the imbalance in cannabis use history between groups reflects ethical and feasibility constraints but limits the ability to disentangle acute intoxication effects from longer-term adaptations associated with habitual use. Third, cannabis exposure was standardized to a single product and dose administered via smoking, which may not reflect the wide variability in cannabis potency, formulations, and routes of administration encountered in real-world settings. Finally, although the performance tasks were selected for their relevance to occupational safety, they cannot fully capture the complexity of real-world job demands or environmental stressors.

In conclusion, regular cannabis users in the experimental group (smoking or ingesting edibles three or more times weekly over the last six months) who consumed cannabis during the study experienced specific, measurable changes in physical performance outcomes up to 12 h post-consumption, even when subjective intoxication feelings were minimal. These impairments were task-specific, affecting precision, sustained submaximal forces, and rapid force development. While not all performance outcomes were significantly impaired, individuals should consider avoiding physically demanding tasks involving precision or endurance for at least 12 h post-consumption. From a public health and workplace safety perspective, these findings have important implications for impairment education, return-to-work guidance, and policy development in safety-sensitive occupations, particularly in contexts where subjective intoxication or biological markers are used as proxies for functional capacity. Future research should prioritize larger and more diverse samples, direct comparisons between frequent and infrequent users, evaluation of alternative routes of administration and higher-potency products, and the development of task-specific functional impairment assessments that better reflect real-world occupational demands.

## Ethics Approval and Consent to Participate

The experimental protocol and consent form was emailed initially and then verbally explained to all participants upon arrival to the first session. Participants then read and signed the informed consent form prior to experimentation. All data was anonymized such that the researcher could not identify individuals when conducting the analyses. This research was approved by the institution's Human Research Ethics Board (Interdisciplinary Committee on Ethics in Health Research: #2022.157) and conducted according to the latest version of the Declaration of Helsinki. The experiments complied with the current laws of the country in which they were performed.

## Supplemental Material

sj-docx-1-dre-10.1177_00472379261433203 - Supplemental material for Physical Performance Impairment with Cannabis Consumption in Adults Over 12 Hours Supplemental material, sj-docx-1-dre-10.1177_00472379261433203 for Physical Performance Impairment with Cannabis Consumption in Adults Over 12 Hours by Waseem Abu-Ashour, Michael Wahl, Saman Hadjizadeh Anvar, Mohammadmahdi Bahrami, Ali Zahiri, Jose Carlos Aragão-Santos, John T. Weber and David G. Behm in Journal of Drug Education
