# Supplementary material for: Physical Performance Impairment with Cannabis Consumption in Adults Over 12 Hours
Source: J Drug Educ. 2026 Mar 23;55(3):127–51. doi: 10.1177/00472379261433203 (PMC13392165; doi:10.1177/00472379261433203)
Supplement: sj-docx-1-dre-10.1177_00472379261433203 - Supplemental material for Physical Performance Impairment with Cannabis Consumption in Adults Over 12 Hours [file sj-docx-1-dre-10.1177_00472379261433203.docx]

**Supplemental Material A. Detailed Methods**

*Participants*

Based on an “a priori'' statistical analysis (G*power version 3.1.9.2, Dusseldorf Germany) to obtain a small magnitude effect size, it was determined that approximately 18 adult participants were needed to achieve an effect size of 0.3 (theoretical assumption), considering the interaction between two groups and four times, alpha (α) of 0.05 and a power of 0.8. Based on convenience sampling, 28 participants (Experimental group: 16 frequent cannabis users and 12 non-smoking controls) were recruited for this study (Table 1). Participants were considered frequent cannabis consumers, which was defined as smoking or ingesting edibles three or more times per week on average over the last six months. For comparison, McCartney et. (2021) define regular cannabis consumers as individuals who use cannabis on a weekly basis or more. Exclusion criteria included participants with the following: 1) history of neurological or psychiatric disorders, 2) engagement in psychological treatment (within the previous 6 months), 3) current or past diagnoses of psychotic disorders, 4) participants taking medications that could influence psychometric testing, 5) participants with any history of alcohol or other substance use disorder (other than cannabis use disorder) 6) female participants were excluded if they were pregnant, looking to become pregnant, or breastfeeding. Participants were asked to refrain from consuming cannabis 12 hours before testing and during the entire day of testing (post-smoking intervention started at either 9 or 10 AM and final testing was completed at 9 or 10 PM [21:00-22:00]) respectively. All participants completed the experimental timeline.

The experimental protocol and consent form was emailed initially and then verbally explained to all participants upon arrival to the first session. Participants then read and signed the informed consent form prior to experimentation. All data was anonymized such that the researcher could not identify individuals when conducting the analyses. This research was approved by the institution’s Human Research Ethics Board (Interdisciplinary Committee on Ethics in Health Research: #2022.157)) and conducted according to the latest version of the Declaration of Helsinki.

*Experimental Design*

Testing was conducted over a three-day period with each participant involved for a 14-hour period within one day (Figure 1). Testing commenced at either 8 or 9 AM with groups of four individuals in each one-hour session. Testing prior to the intervention (cannabis smoking at either 9 or 10 AM) and post-intervention (post-testing at 1-, 6- and 12-hours post-smoking intervention) involved measures of body mass, height, perceived feelings of intoxication, resting blood pressure (BP) and heart rate (HR), reaction time (RT), stork balance test on a padded mat with eyes open, and on a concrete floor with eyes closed, dominant limb handgrip maximal voluntary isometric contraction (MVIC) strength, maintenance of 10%, 20% and 40% of handgrip MVIC for 10-seconds and handgrip endurance test to task failure (inability to maintain prescribed force for three seconds) at 40% MVIC. Many of the aforementioned measures are crucial in various workplaces where slower reaction times (police, military, truck drivers), poor balance (e.g., construction workers working on roofs, marine environments), impaired strength or endurance could negatively impact work performance in labor intensive occupations (e.g.,decreased endurance necessitate more frequent rest periods or insufficient recovery) or contribute to injuries (e.g., slips or falls due to inadequate balance, overuse injuries due to decreased strength or endurance). Blood, urine, and saliva collection was also conducted by certified nurses at each testing period.

**Figure 1**: Experimental Design.

Definitions: CBD: cannabidiol MVIC: maximum voluntary isometric contraction, THC: Delta-9-tetrahydrocannabinol.

*Independent Variable (Cannabis Intervention)*

Following the pre-tests at 8 or 9 AM, participants were brought to a different location within the facility to smoke a cannabis cigarette (Ezee Chill pre-roll: God Father OG), which was provided by a local consumer company (Atlantic Cultivation Inc.). According to the manufacturer web site (https://www.leafly.ca/strains/godfather-og), this is an indica strain with the effects generally being sedating and relaxing. The pre-rolled cigarette contained 0.35 grams of dried cannabis, with 15.9 mg/g concentration of THC, and 0.9 mg/g concentrations of CBD. Participants were asked to consume the entire study-provided product, if they could, and were instructed to finish it within a 10 to 20-minute timeframe to ensure consistency across participants. After consumption, participants were immediately escorted back to the testing site to begin post-consumption assessments. All participants reported being familiar with the smoked cannabis product. Since they were frequent consumers, the amount consumed was within their normal consumption range, ensuring that the study protocol did not introduce unusual consumption behavior that could skew results. Participants also reported their typical methods of cannabis consumption, which included smoking, vaporizing, oral ingestion, or other forms. This information was collected to contextualize the study's intervention approach and ensure participants' familiarity with the consumption method used in the trial.

Smoking was monitored by a researcher to ensure full inhalation. The researcher also monitored for any adverse reactions (none occurred as they were relatively frequent users). There were also two trained registered nurses on site for further follow up and monitoring of participants at all times. After cannabis consumption, participants returned to the testing site for the remaining testing periods.

*Dependent Variables (Measures)*

*Anthropometric Data*

Body mass was measured using a calibrated commercial scale (Accuweight Moreno Valley, CA 92551, USA), which was calibrated to zero before each measurement session using standard calibration weights to ensure accuracy. The scale had a measurement precision of ±0.1 kg. Height was measured with a commercially available measuring tape that was firmly secured to a flat, vertical wall surface to ensure accuracy. The tape was checked for accuracy against a standardized metal rod prior to the study and re-verified weekly. Measurements were recorded to the nearest 0.1 cm. To minimize error, participants stood upright without footwear, with heels together, and the head positioned back to the wall. Participants were queried regarding age, sex, and activity level, which was defined as either a) sedentary: no regular weekly exercise, b) recreationally active: 2-3 regular exercise sessions per week, or c) trained: >3 training sessions per week. We considered sedentary as engaging in occasional and/or incidental physical activity, such as walking to work, light household activities, or casual walking, but not participating in structured exercise programs. This group includes individuals who do not meet the minimum physical activity guidelines for health benefits. Recreationally active were defined as engaging in at least 150 to 300 minutes of moderate-intensity activity (such as brisk walking, cycling, or swimming) or 75 to 150 minutes of vigorous-intensity activity (such as running, high-intensity interval training) per week. This category also includes muscle-strengthening activities at least two days per week. The primary goal of these activities is to improve general health and fitness, without a competitive or performance-driven focus. Trained were defined as engaging in regular, structured training sessions more than three times per week. Individuals in this category typically identify with a specific sport or athletic discipline and engage in purposeful training aimed at preparing for competition. This includes specialized strength, endurance, or skill-based training programs designed to enhance performance in competitive settings (McKay et al. 2022).

While these anthropometric and demographic measures were not expected to change throughout the study, they were critical for understanding potential baseline differences in physical performance and response to cannabis consumption. For example, body mass can influence how cannabinoids are metabolized and distributed in the body, potentially affecting impairment levels (Rossi et al. 2018, Lucas et al. 2018). Similarly, activity level could moderate performance outcomes, as physically active individuals may display different endurance or balance capabilities. Including these measures allows for better contextualization of the performance results and understanding potential confounding factors related to workplace safety outcomes.

*Blood Pressure (BP) and Heart Rate (HR)*

BP and HR were monitored as prolonged and consistent elevation of these cardiovascular parameters are indicators of increased stress (physical or mental) and could adversely affect health and quality of life (Liu et al. 2017, Virginia de Carvalho 2013). Participants were requested to remain seated and relaxed for a 2-minute period before the blood pressure device (Omron Healthcare, Inc. 2-3-13 Konan, Minato-ku, Tokyo Shinagawa Front Building 7F) was initiated (Brady et al. 2021). While seated and relaxed, their skin was prepared and electromyography (EMG) electrodes were placed over the forearm flexors (more detail provided in the EMG section). The cuff was placed on the left arm (heart is situated slightly to the left of the midline) directly superior to the cubital fossa with the arm relaxed and extended with the palm supinated and the arm resting on a table at shoulder height. The BP device automatically inflated and deflated and provided the systolic and diastolic BP as well as HR.

*Subjective Perceived Feelings of Intoxication*

Participants were asked while relaxing prior to the BP measurement about their perceived level of intoxication. They were asked to mark a line on a 10 cm horizontal line that was designated from 0 -100%. They were asked how “high” or “stoned” they felt with zero being absolutely no feeling of intoxication while 100% was the most “stoned” or “high” (intoxicated or inebriated) they had ever felt. The terms “stoned” or “high” were used as these words are common vernacular among these individuals.

*Reaction time (RT)*

RT was measured on a laptop computer (Dell Lenovo computer) using a web application (Web site: faculty.washington.edu/chudler/java/redgreen.html). The participant had to tap the trackpad as quickly as possible when a green light appeared on a simulated traffic light. Five attempts were permitted and the average of the five attempts was used for analysis. If one measure was considered an outlier (e.g., more than 100 ms slower than the other four attempts), it was not considered in the average. If more than one attempt was considered an outlier then another five attempts were conducted. As stated, five attempts were averaged with outliers excluded; however, no single participant’s data were excluded from the analysis. There were only two occasions in which a participant was distracted by another individual and did not successfully complete a reaction time attempt. These instances were readily identifiable, as typical reaction times of a few hundred milliseconds increased to approximately 2–3 seconds when distraction occurred. In these cases, the participant did not respond to the initial appearance of the green light, meaning that a true reaction time was not recorded. When this occurred, the attempt was repeated immediately to ensure that the recorded values reflected valid responses to the stimulus rather than attentional distraction. The day-to-day reliability of the RT using an intraclass correlation coefficient (ICC) with prior testing from this laboratory group has been reported to be between 0.79 and 0.93 (Behm et al. 2004).

*Static Stork balance test*

Two trials each of the Static Stork balance test were conducted with eyes open and eyes closed with no shoes (stocking feet). With eyes open, the participant stood on a 45 cm^2^ square foam pad with a thickness of 10 cm. The eyes closed Static Stork balance test was conducted on a concrete floor. While standing, the participant placed their hands on the hips, then they positioned the non-supporting foot against the inside knee of the supporting leg. The participant was provided one minute to practice the balance test. The dependent variable was time which was monitored with a stopwatch. Timing began when the non-supporting foot was placed against the inside (approximately knee height) of the supporting leg. The timing was stopped if a) hand(s) came off the hips b) supporting foot swiveled or moved (hops) in any direction, or c) non-supporting foot lost contact with the knee. The two trials of the balance tests were conducted on both legs. The longest time was used for analysis. Reliability has been reported to be excellent with ICCs ranging from 0.91 to 0.93 (Aranha 2019).

*Handgrip* *Maximum Voluntary Isometric Contractions (MVIC)*

While standing, participants gripped a custom designed (Technical Services of Memorial University of Newfoundland) isometric handgrip device with their dominant hand (hand used for writing). They initially performed two handgrip MVICs and if the second MVIC was more than 5% greater than the first MVIC then a third MVIC was performed. One minute recovery was provided between MVICs. The steel bar grip was attached to a Wheatstone bridge strain gauge (Omega Engineering Inc., LCCA 250, St. Eustache, Quebec, Canada). During the 4-second MVICs, the participant would squeeze the handgrip dynamometer as hard and fast as possible with the arm situated away (abducted) from the body to prevent bracing. Researchers provided consistent verbal encouragement; vocalizing “harder” three times within the 4-second MVIC.

Peak force and rate of force development from 0-50, 50-100, 100-150, and 150-200 ms were recorded. The differential voltage (±0.03% linearity and 3 mv/V) from the handgrip dynamometer was calibrated to Newtons and all forces detected by the handgrip dynamometer strain gauge were amplified (500x) (Biopac Systems Inc., DA 150), and analog to digital converter (MP150WSW) and monitored on computer. All data was stored on a computer at a sampling rate of 2000 Hz. Data was recorded and analyzed with a commercially designed software program (Acq-Knowledge III, Biopac Systems Inc.). This laboratory has previously reported excellent intersession reliability (ICCs of 0.98-0.99) using these techniques (Behm et al. 1996; Behm et al. 2002; Low et al. 2019).

*Movement (Submaximal Force) Control*

Two minutes following the MVIC, the participants were tested for movement control (isometric force steadiness) by viewing a computer monitor and maintaining relative forces of 10-, 20- and 40% of MVIC for 10-seconds each. One minute of recovery was allocated between each contraction intensity. In order to minimize the possibility of fatigue, the order was not randomized as the 10% and 20% MVICs were considered very light or low intensity contractions.

The data (6-second period commencing 2-seconds after the start of the contraction and ending 2-seconds before the end of the contraction) was analyzed for the average relative deviation from the prescribed force ((prescribed force - average force) x 100 = % difference from prescribed force)). The same time period was analyzed for the standard deviation (SD) of the force as a measure of force output variability. Prior research from this laboratory has reported day-to-day reliability (intraclass correlation coefficient: ICC) of the matching force test to be 0.8, with a between test (single session) reliability of 0.88 (Behm et al. 2004).

*Handgrip Endurance*

The 40% handgrip MVIC was maintained until task failure. The first 10-seconds was analyzed for the movement control measures but the participant then continued to maintain the relative force till exhaustion (task failure). Task failure was defined as the inability to maintain the prescribe force (second time the participant could not maintain the prescribed force for 3-s). Maximum endurance time was recorded.

*Electromyography (EMG)*

Surface EMG (s-EMG) was used in this study to record muscle activity of the forearm and finger flexors. Muscle strength, endurance, and control of the wrist and fingers is ubiquitous in many professions whether the individual works in an office on a computer keyboard or with more physically stressful occupations (e.g., construction, driving, military, industrial work lines and many others). A number of forearm flexor muscles are involved in wrist stabilization and finger flexion with the handgrip (e.g., flexor carpal radialis, flexor carpal ulnaris, flexor pollicis longus muscle, flexor digitorum profundus, flexor digitorum superficialis, and palmaris longus muscles) and located generally on the palmar side of the forearm (Basmaijan 1981). Before electrodes were placed on the skin, investigators prepared the area by shaving, abrading, and cleaning the skin with an isopropyl alcohol swab before letting it dry (Hermens et al. 1999). Self-adhesive Cl/AgCl bipolar electrodes (MeditraceTM 130 ECG conductive adhesive electrodes, Syracuse, USA) were used in parallel with the muscle fibers and systematically placed according to SENIAM guidelines (Hermens et al. 1999). The SENIAM guidelines are a standardized internationally recognized protocol developed to improve the reliability and reproducibility of surface electromyography (s-EMG) recordings by ensuring consistent electrode placement, reducing variability, and minimizing crosstalk from adjacent muscles. Following these guidelines, electrodes were placed parallel and edge-to-edge for an inter-electrode spacing of 20 mm, ensuring consistent alignment with the muscle fibers to maximize signal accuracy. Specifically, the electrodes were placed at 80% of the longitudinal distance from the radial head to the medial epicondyle of the humerus and 80% of the lateral to medial side distance of the palmar forearm at that location (medial side). The ground electrode was placed on the medial epicondyle of the humerus, and all leads were taped to the skin to help minimize any movement artifacts in the s-EMG signal. EMG signals were amplified 1000x and filtered with a 3-pole Butterworth filter with cut-off frequencies of 10-500 Hz (bi-polar differential amplifier, input impedance = 2MΩ, common-mode rejection ratio > 110 dB min (50/60 Hz), noise > 5 µV), (Biopac Systems Inc., DA 150, and analog to digital converter (MP150WSW)). Analog signals were digitally converted at a sampling rate of 5 kHz and sampled at 2000 Hz (Biopac Systems Inc., DA 150, and analog to digital converter (MP150WSW). EMG integral and the mean amplitude of the root mean square (RMS) were measured over a 1-second window to capture a measure of muscle activation during the peak force (500 ms before and 500 ms after the peak force output). The median frequency was obtained from a scan that included 5-250 Hz. These EMG parameters were also monitored for the middle 6-seconds of the 10-, 20- and 40% MVIC movement control contractions. Finally, during the 40% MVIC endurance (fatigue) protocol, these EMG measures were analysed for each quartile of the handgrip duration. A commercial software program (AcqKnowledge III, Biopac Systems Inc., Holliston, MA) was used to analyze the digitally converted analog data.

*Cannabinoid Testing:*

This study investigated the temporal pattern of THC and Carboxy-THC levels in blood and urine samples following cannabis consumption at baseline and at 1-, 6-, and 12-hours post-consumption. The analyses were aimed at understanding the pharmacokinetic profiles of THC and its primary metabolite, Carboxy-THC, which is known for its longer detection window in the body (Huestis and Smith, 2018). We used oral fluid, urine, and blood tests to detect THC levels in recruited participants. A registered practicing nurse was responsible for collecting oral fluid, urine and blood samples. This was conducted at baseline (pre-consumption), 1 hour, 6 hours and 12 hours post-consumption of cannabis. Moreover, the nurse monitored the symptoms and to ensure if further medical attention is required. The samples were stored in a university laboratory freezer at the temperature of -80°C until shipping.

Blood Cannabinoids Lab Processing:

Extraction and analysis of THC, THCA, OH-THC, CBD, and CBDV in whole blood was performed according to a method developed in-house by Dynacare labs® (Ottawa, Ontario, Canada), a certified research analysis laboratory specializing in different product sample testing. Prior to sample collection, consultation with Dynacare Labs® was conducted to ensure that the most appropriate collection, handling, storage, and processing protocols were followed to ensure sample integrity. Based on their recommendations, blood samples were collected using BD Vacutainer® K2 EDTA 4 mL tubes, which are specifically designed for whole blood collection. To minimize the risk of cannabinoid adherence to the plastic surfaces the lab advised prompt processing of samples immediately after collection. Samples were handled, stored, and processed following their standardized protocols to ensure consistency and prevent degradation. Briefly, 100 µL of each sample was crushed and mixed with methanol containing the Cannabinoids Working Internal standard. Samples were vortexed for 60 seconds, then allowed to equilibrate at room temperature for 10 minutes. For both blood and urine samples the following procedure was the same. Subsequently (after the 10 minutes of equilibration for blood and the 60 seconds of urine vortex respectively), samples were centrifuged at 4500 RPM for 5 minutes. Supernatant was transferred into an HPLC vial and injected onto the Prominence HPLC System (Shimadzu) followed by subsequent analysis on the 6500+ QTRAP LC-MS/MS (SCIEX). All analytical data were collected and processed. The concentration of cannabinoids in the samples was determined using linear regression with a weighting factor of 1/x. The limit of quantitation (LoQ) for all cannabinoids was 2 ng/mL, with an analytical measuring range of 2.0 to 5000 ng/mL.

Urine Cannabinoids Lab Processing:

Extraction and analysis of THC, THCA, OH-THC, CBD, CBDA and OH-CBD in urine was performed according to a method developed in-house by Dynacare labs®. Briefly, 100 µL of each sample along with the addition of Cannabinoids Working internal Standard (IS) in methanol, was hydrolyzed using both enzyme and alkaline digestion method. After hydrolysis, the sample was neutralized and vortexed for 60 seconds. As mentioned in the previous section, subsequent analysis was the same for blood and urine.

Saliva Test:

The VeriCheck® Oral Fluid THC Test was utilized to measure THC levels in the participants’ saliva. This single-use, one-step test kit is designed for on-site, instant screening, advertised to detect THC concentrations as low as 10 ng/mL (https://www.drugtestkits.ca/vericheck-oral-fluid-saliva-thc-test). The test does not require any additional sponges or activation steps, and it includes an integrated flow line to ensure that a sufficient quantity of oral fluid is collected.

*Statistical Analyses*

Data were analyzed using the statistical software Jamovi (The Jamovi Project (2022), Jamovi, Version 2.3.18.0 (1) <https://www.jamovi.org>.) All the continuous characterization data were presented with marginal means and standard deviations and compared using a one-way ANOVA. The categorical characterization data were shown based on absolute and relative frequency and compared using the chi-square test. The inferential statistics were given based on regression estimates and a 95% confidence interval (CI).

For physical performance measures, generalized linear mixed models were used. Initially, a visual inspection of the data based on histogram graphs was conducted to see the symmetry of the data. Since all the outcomes were continuous variables, the data were analyzed using the Gamma distribution that fits asymmetric data better. The group (i.e., experimental vs. control) and time (i.e., pre-test, 1-, 6-, and 12-hours) and the interaction effect (group × time) were defined as fixed effects. Besides, the participants’ intercepts were used as a random effect for addressing individual variations in the repeated measures model.

When the interaction effect was detected, the estimates were analyzed using the pre-test and the control group as reference levels. Based on the specific interaction regression estimates and CI, the observed effects were described based on the variations. All comparisons adopted a significance level of p < 0.05. Beta (β) scores represent the variation in the experimental group vs. the variation in the control group between pre-intervention and 1h.

The effects of time and treatment on THC metabolite concentrations were statistically analyzed using a two-way Analysis of Variance (ANOVA). The factors analyzed were 'time post-consumption' as a within-subject factor and 'treatment' (placebo vs. cannabis) as a between-subject factor. Given the repeated measures design, the assumption of sphericity was verified using Mauchly's test of sphericity. Where sphericity was not assumed, degrees of freedom were corrected using Greenhouse-Geisser estimates. Post hoc comparisons were conducted using the Bonferroni correction method to adjust for multiple testing and control type I error, with the level of significance set at p < 0.05.
